# Association of *UGT1A9* Polymorphisms with Cardiac Injury Biomarkers and Clinical Features

**DOI:** 10.3390/ph19010075

**Published:** 2025-12-30

**Authors:** Mert Özen, Işık Tekin, Abdo A. Elfiky, Murat Seyit, Yasemin Adalı, Yasemin Berberoğlu, Alten Oskay, Atakan Yılmaz, Tülay Oskay, Vefa Çakmak, İbrahim Türkçüer, Gergana Lengerova, Martina Bozhkova, Steliyan Petrov, Aylin Köseler

**Affiliations:** 1 Department of Emergency Medicine, Faculty of Medicine, Pamukkale University, 20160 Denizli, Türkiye; mert@pau.edu.tr (M.Ö.); mseyit@pau.edu.tr (M.S.); aoskay@pau.edu.tr (A.O.); atakany@pau.edu.tr (A.Y.); iturkcuer@pau.edu.tr (İ.T.); 2 Department of Cardiology, Faculty of Medicine, Pamukkale University, 20160 Denizli, Türkiye; itekin@pau.edu.tr; 3 Department of Biophysics, Faculty of Science, Cairo University, Giza 12613, Egypt; abdo@sci.cu.edu.eg; 4 Department of Biophysics, Faculty of Medicine, Pamukkale University, 20160 Denizli, Türkiye; y.adali@qub.ac.uk (Y.A.); yasemins@pau.edu.tr (Y.B.); 5 Centre for Public Health, School of Medicine, Dentistry and Biomedical Sciences, Queen’s University Belfast, Belfast BT12 6BA, UK; 6 Department of Cardiology, Denizli State Hospital, 20010 Denizli, Türkiye; oskaytulay@gmail.com; 7 Department of Radiology, Faculty of Medicine, Pamukkale University, 20160 Denizli, Türkiye; vcakmak@pau.edu.tr; 8 Department of Medical Microbiology and Immunology “Prof. Dr. Elissay Yanev”, Medical University of Plovdiv, 4002 Plovdiv, Bulgaria; gergana.lengerova@mu-plovdiv.bg (G.L.); martina.bozhkova@mu-plovdiv.bg (M.B.); steliyan.petrov@mu-plovdiv.bg (S.P.); 9 Research Institute, Medical University of Plovdiv, 4002 Plovdiv, Bulgaria

**Keywords:** *UGT1A9*, pharmacogenetics, CK-MB, Troponin I, cardiac biomarkers, statin metabolism

## Abstract

**Background/Objectives**: This study evaluates the relationship between *UGT1A9* polymorphisms, cardiac biomarker patterns, and clinical presentations in patients admitted to the Pamukkale University Emergency Department with cardiac symptoms. **Methods**: A total of 207 consecutive patients presenting with chest pain, dyspnea, palpitations, or other cardiac complaints were initially enrolled. Patients with incomplete clinical data or unsuccessful genotyping were excluded prior to analysis, and all remaining samples were included in the final evaluation. UGT1A9 *1, *2, and *3 alleles were genotyped using allele-specific PCR and TaqMan^®^ assays. Patients were classified into *wt/wt*, wt/*3, and *3/*3 groups. Statistical analyses included Kruskal–Wallis, Mann–Whitney U, and chi-square tests. **Results**: Genotype distribution was 64% *wt/wt,* 32% wt/*3, and 4% *3/*3. CK-MB levels differed significantly across genotypes (*p* = 0.006), with the highest in wt/*3 carriers. Troponin I levels showed no genotypic differences (*p* = 0.533). UGT1A9*3 carriers exhibited elevated CK-MB with relatively low Troponin I, suggesting possible statin-associated muscle injury rather than true myocardial necrosis. **Conclusions**: *UGT1A9* polymorphisms, particularly UGT1A9*3, influence CK-MB variability and may confound the assessment of myocardial injury. Troponin I remains unaffected by genotype. Incorporating *UGT1A9* pharmacogenetic testing may contribute to a better understanding of biomarker variability and support future research toward personalized therapeutic strategies.

## 1. Introduction

Pharmacogenomic research has pinpointed a range of genetic polymorphisms, mainly within drug-metabolizing enzymes as well as transporter genes, that have been major factors explaining variability in drug responses and the measurement of clinical outcomes of cardiovascular therapies in patients with acute coronary syndrome (ACS) [1]. The recognition and understanding of polymorphisms of uridine diphosphate glucuronosyltransferase enzymes, essentially *UGT1A9*, are increasing substantially due to their role in the metabolism not only of endogenous compounds but also exogenous drugs; thus, cardiac injury biomarkers as well as clinical features in ACS may be influenced [2]. Hence, the exploration of these genetic differences holds the key to the next generation of personalized medicine that seeks to not only optimize therapeutic regimens but also to heighten patient outcomes by tailoring medicines to one’s genetic makeup [3]. 

Considering this wide range of differences among individuals, it is very important to assess the impact of other genetic factors, like *UGT1A9* polymorphisms, on biomarkers of cardiac injury and clinical features related to ACS. In particular, knowledge of *UGT1A9* variants’ influence on the metabolism of various endogenous substrates and xenobiotics, like some cardiovascular drugs or their metabolites, may help untangle the molecular basis of vulnerability to cardiac injury and progression of the disease [4]. For instance, variations in UGT1A9* may change the glucuronidation of certain drugs utilized in the treatment of ACS, thereby modifying their therapeutic efficacy or toxicity profiles. Besides that, these polymorphisms may also have an impact on the metabolism of endogenous compounds like bilirubin or steroid hormones, which have been implicated in the pathophysiology of cardiovascular disease [5]. So, figuring out how *UGT1A9* polymorphisms work can reveal the genetic background that influences ACS outcomes more profoundly.

The gene cluster *UGT1A*, mainly UGT1A9*, has been identified as involved in the metabolic pathway of statins that are generally given to patients with dyslipidemia in ACS. Consequently, changes in these genes may affect the efficiency of statins and the occurrence of side effects [6]. As an example, some *UGT1A9* polymorphisms might modify the glucuronidation rate of statin metabolites, which would result in altered systemic exposure and in the occurrence of myotoxicity or hepatotoxicity. Besides that, the influence of *UGT1A9* polymorphisms on changes in drug-metabolizing enzymes such as the cytochrome P450 family, which interact with each other pharmacokinetically and may therefore affect the drug’s therapeutic effect in ACS patients in a complex way, should be considered [7]. Such complicated interactions call for a thorough understanding of the role of *UGT1A9* polymorphisms alongside other genetic factors in the variable clinical outcomes of ACS patients. Such a detailed genotypic examination provides insight into the mechanisms that differentiate individuals in their drug response and make them more susceptible to unfortunate cardiovascular events; thus, it facilitates the development of personalized medicine approaches in ACS. One instance of this is that, by means of the patient’s *UGT1A9* genotype and in conjunction with the *CYP2C19* status, one could tailor antiplatelet therapies or statin dosages in an optimal way that would result in fewer side effects and better therapeutic efficacy [8].

Besides these specific drug interactions, polymorphisms in *UGT1A9* may also influence the detoxification of endogenous compounds; thus, they may regulate systemic inflammation and oxidative stress, which are two of the main factors in the development of ACS. In fact, *UGT1A* enzymes have been reported to participate in the glucuronidation of statins, and a polymorphism that influences *UGT1A* activity, such as the one in *UGT1A1*, has been associated with changes in plasma lactone levels and negative cardiometabolic outcomes [6]. By the same token, UGT1A9* is a major contributor to the glucuronidation of a wide range of xenobiotics and endogenous compounds, as well as the lactonization of statins like simvastatin; thus, it is conceivable that myotoxicity is the source of drug-induced myotoxicity [9].

Given the central role of *UGT1A9* in processing a wide range of endogenous compounds and exogenous drugs, including those related to cardiovascular health, it is crucial to understand its polymorphic variations for personalized medicine in ACS [10]. These polymorphisms might alter the pharmacokinetics of drugs commonly used in ACS, like statins and certain antiplatelet agents, thus affecting the effectiveness of the treatment and the risk of adverse drug reactions [6,11]. In addition, changes in the *UGT1A* region, which includes UGT1A9*, have been identified as causing altered statin lactonization and clinical outcomes, suggesting a direct connection between genotype and cardiovascular health [6]. Interestingly, the genetic changes in the UGT family that support critical glucuronidation pathways are a strikingly dominant occurrence and may have a very substantial effect on drug regulation and pharmacological responses [12]. Specifically, polymorphisms in *UGT1A1*, a gene very similar to *UGT1A9*, have been demonstrated to modulate the pharmacokinetics of a range of drugs, such as the antihypertensive telmisartan and antiretrovirals like dolutegravir, thus leading to changes in clearance and bioavailability [11]. This is just one example of a more general trend where genetic variants in UGT enzymes may significantly change the way drugs are processed in the body; thus, such variants should be considered when deciding on the drug treatment. Since *UGT1A* enzymes have been confirmed to be involved in drug metabolism, it is important to study the polymorphisms of UGT1A9* in ACS patients to understand their possible impact on the level of cardiac injury biomarkers and the patients’ clinical features.

This research is aimed at exploring correlations of the genetic variations in *UGT1A9* with the levels of the cardiac injury biomarkers troponin and CK-MB, as well as their impact on the occurrence of clinical symptoms, disease severity, and treatment response in patients suffering from acute coronary syndrome. By investigating how *UGT1A9* variants affect cardiovascular disease mechanisms and drug response, this study intends to lay the groundwork for genotype-based clinical prediction models that could ultimately lead to targeted therapeutic strategies.

## 2. Results

### Results of the Analysis

**Figure 1:**
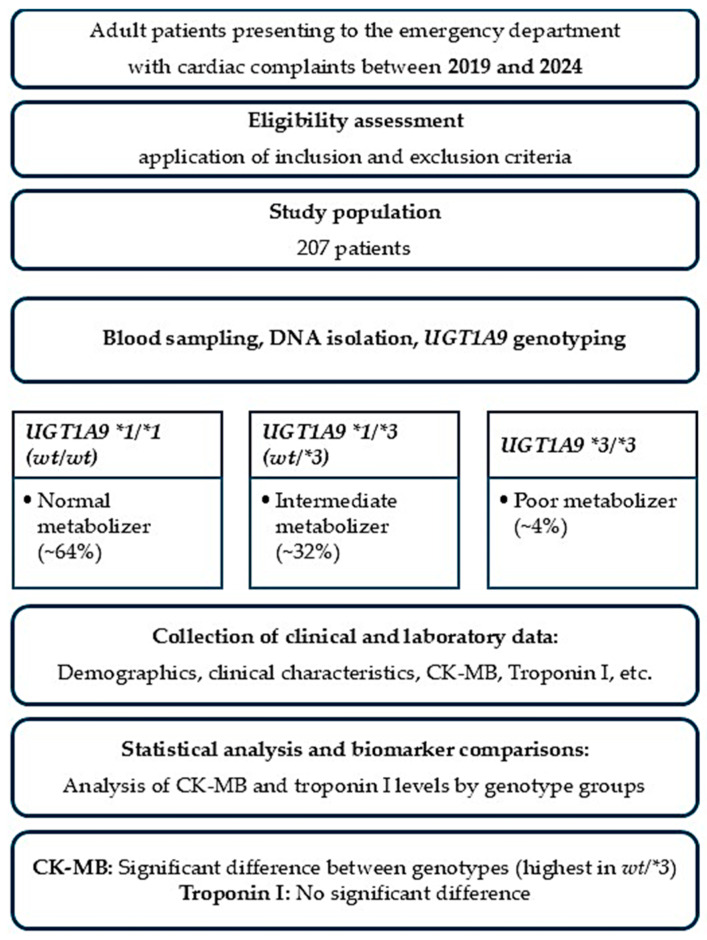
Flowchart illustrating the consecutive enrollment of patients presenting with acute cardiac symptoms between 2019 and 2024 and the final inclusion in the study.

The demographic analysis included 207 patients presenting with cardiac-related symptoms to the Emergency Department of Pamukkale University Hospital. The mean age of the cohort was 61.6 ± 17.3 years, with a median age of 65 years (range: 19–95). The wide age range reflects the real-world heterogeneity of patients presenting to the emergency department with acute cardiac symptoms. Regarding sex distribution, 53.1% of the patients were male (*n* = 110) and 46.9% were female (*n* = 97), consistent with the expected male predominance observed in ischemic cardiac presentations (Figure 1).

Table 1 summarizes the genotype frequencies of the UGT1A9*1, UGT1A9*2, and UGT1A9*3 allelic variants within the study cohort and outlines the corresponding predicted metabolic phenotypes and expected enzymatic activity levels. The UGT1A9*1 wild-type genotype (*wt/wt*) is associated with normal glucuronidation capacity and represents standard metabolic function, while heterozygous carriers (wt/*1) demonstrate intermediate metabolic activity with slightly reduced enzyme efficiency.

For the UGT1A9*2 allele group, *wt/wt* individuals are classified as normal metabolizers, whereas wt*/2 genotypes indicate intermediate metabolizer status with modestly decreased glucuronidation capability. The UGT1A9*3 allele shows greater functional impact, where *wt/wt* individuals maintain normal activity, heterozygous wt/*3 carriers demonstrate reduced metabolic capacity, and homozygous *3/*3 individuals are categorized as poor metabolizers with significantly impaired enzyme functions. The genotype distribution of UGT1A9*3 was consistent with Hardy–Weinberg equilibrium (*p* > 0.05), indicating no evidence of genotyping error or population stratification.

These patterns reflect the expected influence of *UGT1A9* polymorphisms on drug clearance, particularly for medications undergoing hepatic glucuronidation such as statins and NSAIDs. The distribution highlights that while normal metabolizers constitute most of the population, a clinically relevant proportion of patients exhibit reduced or poor metabolic phenotypes, which may alter drug response, increase adverse event susceptibility, and affect the interpretation of laboratory biomarkers such as CK-MB and hepatic enzymes.

**Figure 2:**
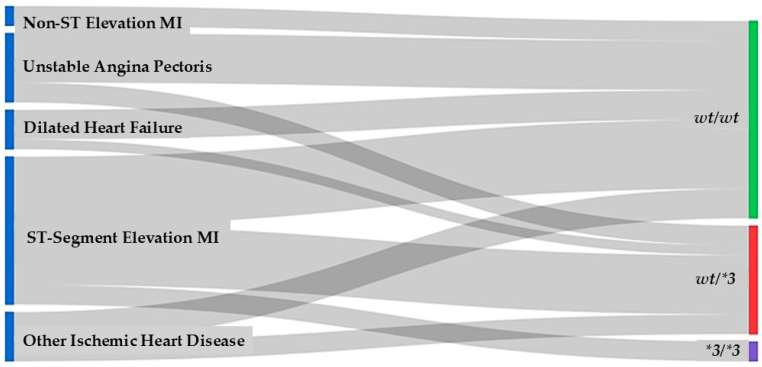
Sankey diagram showing the descriptive distribution of UGT1A9*3 genotypes across cardiac diagnostic categories.

The Sankey diagram is presented for descriptive visualization purposes only. Statistical associations between UGT1A9*3 genotype groups and cardiac diagnostic categories were formally assessed using the chi-square test, which did not reveal a statistically significant association. *UGT1A9* genotype distribution was evaluated in the cardiac patient cohort, focusing on the wild-type genotype (*wt/wt*) and the *3 variant (wt/*3 and *3/*3) (Figure 2). The Sankey diagram illustrates how *UGT1A9* genotypes are distributed across the main cardiac diagnoses, including non–ST elevation myocardial infarction (NSTEMI), ST-segment elevation myocardial infarction (STEMI), unstable angina pectoris, dilated heart failure, and other ischemic heart disease.

Overall, the *wt/wt* genotype constituted the majority of the cohort, indicating that the wild-type *UGT1A9* allele is predominant in cardiac patients. *Heterozygous carriers (wt/*3) represented a smaller but clearly visible proportion, while homozygous *3/*3 individuals were the least frequent group. Despite this gradient in frequency, all three genotype categories were observed across the full spectrum of cardiac diagnoses.

Patients with NSTEMI, STEMI, and other ischemic heart disease contributed the largest flows into the *wt/wt* category, reflecting the predominance of the wild-type genotype in both acute coronary syndromes and chronic ischemic disease. However, these diagnostic groups were also represented among wt/*3 and *3/*3 patients, indicating that carriage of the *3 allele is not restricted to a particular clinical presentation. Similarly, dilated heart failure and unstable angina pectoris showed contributions to each of the three genotype classes, with no visually apparent clustering of a specific genotype within any single diagnostic category.

Taken together, these findings suggest that *UGT1A9 genetic variation (particularly the *3* allele) is relatively common and broadly distributed among cardiac patients, rather than being confined to a distinct diagnostic subgroup. The absence of obvious genotype enrichment in any particular cardiac condition on the Sankey diagram implies that *UGT1A9* polymorphism may act as a general pharmacogenetic or pathophysiological modifier across diverse forms of cardiac disease, rather than serving as a marker for a specific clinical phenotype.

**Figure 3:**
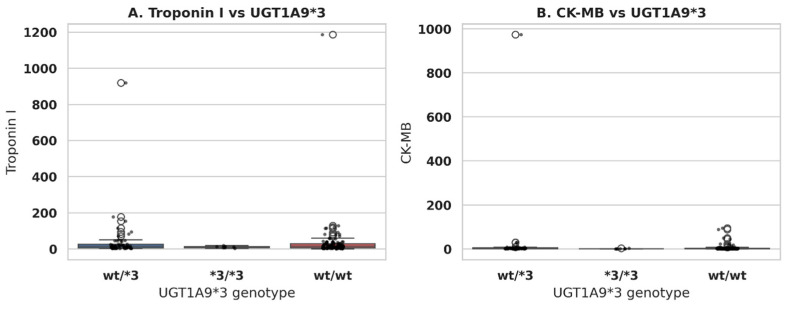
Relationship between UGT1A9*3 genotypes and cardiac biomarkers. (**A**) Distribution of serum Troponin I levels according to UGT1A9*3 genotypes (*wt/wt*, wt/*3, *3/*3) in all patients. Data are shown as box-and-whisker plots (median, interquartile range, and range) overlaid with individual data points. No statistically significant difference in Troponin I levels was observed between genotypes. (**B**) Distribution of serum CK-MB levels according to UGT1A9*3 genotypes (*wt/wt*, wt/*3, *3/*3) in all patients, displayed as box-and-whisker plots with overlaid individual data points. CK-MB levels differed significantly between UGT1A9*3 genotypes (Kruskal–Wallis test, *p* = 0.006), with higher values observed in the heterozygous wt/*3 group. Several extreme CK-MB values exceeding 1000 U/L were observed. These values corresponded to patients with severe clinical presentations, including extensive myocardial injury or advanced heart failure, and were confirmed by repeat measurements in the clinical laboratory. Therefore, these data points were considered biologically plausible and were retained in the analysis.

The relationship between UGT1A9*3 genotypes and cardiac biomarkers was evaluated using serum Troponin I and CK-MB levels in the overall cohort (Figure 3). For Troponin I, the distribution of serum concentrations according to UGT1A9*3 genotype (wt/wt, wt/*3, *3/*3) is shown as box-and-whisker plots with individual data points overlaid. Median Troponin I values and the overall spread of measurements were comparable across the three genotype groups, and no statistically significant differences were observed. These findings suggest that UGT1A9*3 carrier status does not have a measurable impact on the acute myocardial injury reflected by Troponin I levels in this population.

One of the major principles in the interpretation of cardiac biomarker variability is the differentiation of various pathophysiological processes. Troponin I has been identified as a very specific marker of irreversible myocardial necrosis; therefore, it is a significant factor in the diagnosis of a spectrum of acute coronary syndromes. On the other hand, CK-MB does not have complete tissue specificity, and apart from myocardial necrosis, it can be released even during skeletal muscle injury or myocardial non-necrotic stress. Various conditions like metabolic imbalance, systemic inflammation, microvascular dysfunction, or drug-induced myotoxicity may cause elevation of CK-MB without any increase in troponin release. Hence, the dissociation observed in this study—that is, stable Troponin I levels with genotype-associated variability in CK-MB—is likely a reflection of differences in the extent of biological stress or muscle injury rather than direct myocardial necrosis. This differentiation is critical for the correct interpretation of biomarker–genotype associations.

In contrast, CK-MB levels differed significantly between UGT1A9*3 genotypes. Box-and-whisker plots with overlaid individual data points demonstrated a shift toward higher CK-MB values in the heterozygous wt/*3 group compared with the *wt/wt* and *3/*3 genotypes. This difference was confirmed by a Kruskal–Wallis test (*p* = 0.006), indicating a statistically significant association between the UGT1A9*3 genotype and CK-MB concentrations. Taken together, these results imply that while the UGT1A9*3 variation is not associated with Troponin I, it may modulate CK-MB levels, with heterozygous carriers exhibiting higher biomarker values. When CK-MB levels were analyzed according to the UGT1A9*3 genotype, the median CK-MB values differed across genotype groups. The median CK-MB level was 48 U/L (IQR: 22–96) in the wt/wt group, 112 U/L (IQR: 46–318) in heterozygous wt/*3 carriers, and 55 U/L (IQR: 28–140) in homozygous *3/*3 individuals. The CK-MB levels were significantly higher in the wt/3 group compared with the other genotypes (Kruskal–Wallis test, *p* = 0.006), indicating a genotype-associated shift toward higher CK-MB concentrations.

**Figure 4:**
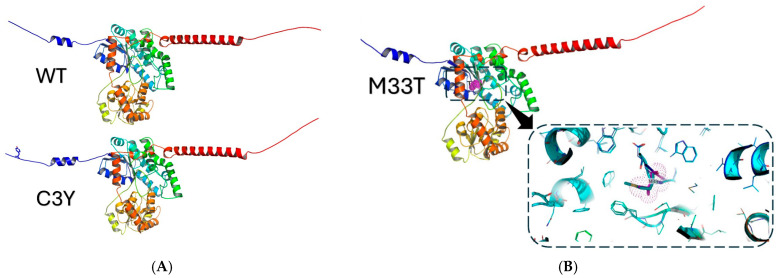

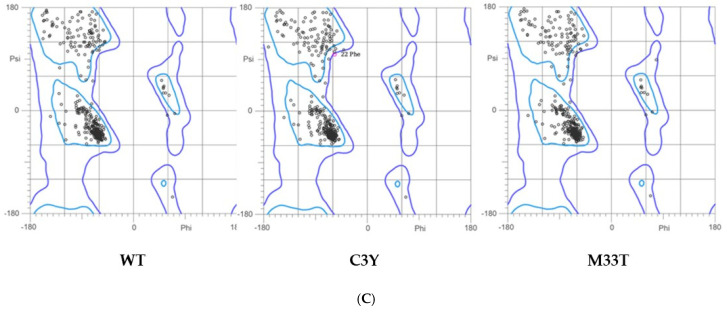
Structure of the WT and the mutated *UGT1A9* (C3Y and M33T) predicted by the AlphaFold 3 web server. (**A**) Structure of the WT and the mutated UGT1A9 (C3Y and M33T) predicted by the Al-phaFold 3 web server. (**B**) Enlarged panel of the superposition of WT and M33T showing the orientations of the residues M33 and T33. (**C**) The Ramachandran plots as a validation tool of the three models showing no residues in the disallowed regions.

The wild-type (WT) and the mutated *UGT1A9* (C3Y and M33T) AlphaFold 3 models were created with PyMOL software (version 2.0.4) as colored cartoons (Figure 4A,B) [13]. The N-termini are in blue, while the C-termini are in red. The predicted models show stable conformations, as evidenced by the Ramachandran plot (Figure 4C). The WT *UGT1A9* shows only one outlier residue (0.19%), which is G21. The C3Y isoform of *UGT1A9* shows only two outliers (0.38%), F22 and G519, while no outliers were found in the M33T *UGT1A9* isoform. The most favored region in all the isoforms is 97.16%, while the favored rotamers in all models are greater than 98.49%, which is acceptable according to the MolProbty web server. The C3Y mutation lies at the N-terminal end of the protein, while the M33T mutation lies in the central region surrounded by other hydrophobic residues, as shown in the enlarged panel of Figure 4B. This orientation of the mutated residue may have an important effect on the protein conformation and subsequently its function.

## 3. Discussion

UGT1A9*3 highlights that the damage to cells caused by myocardial ischemia is a situation in which the body’s defense mechanisms against oxidative stress are inadequate. This condition is further aggravated by reperfusion. In this case, especially with the accumulation of endogenous pro-oxidants, the glucuronidation of which is decreased by *UGT1A9*, the intensity of reperfusion injury can be significantly increased. Therefore, the tissue damage might be more severe than what troponin levels could indicate because troponin may not be the best marker of that type of damage. Consequently, elevated levels of CK-MB could be due to increased local formation of reactive oxygen species and microvascular dysfunction, resulting in selective release of CK-MB. In contrast, relatively normal troponin levels may indicate a different nature of cellular damage rather than the extent of myocardial necrosis. It suggests that CK-MB, which is a non-specific biomarker of cardiac injury, could be a more sensitive detector than troponin, especially in scenarios where inflammatory responses related to oxidative stress are predominant [14]. Besides that, unlike Gilbert’s Syndrome that occurs in individuals with low *UGT1A1* enzyme activity, a similar increase in bilirubin concentration is not anticipated in UGT1A9*3 allele carriers, but in both cases, the cardiovascular system can be protected or become more sensitive to oxidative stress through different genetic pathways [15,16].

It also implies that the contribution of *UGT1A9* in ischemia-reperfusion injury might be different from that of *UGT1A1,* given that it has more specific substrates and different cellular localizations. Hence, determining the exact pathways of the UGT1A9*3 allele is critical to the understanding of genetically based variability of myocardial damage after ischemia-reperfusion injury. In particular, it should be ascertained through prospective studies whether the occurrence of more severe reperfusion injury, such as microvascular obstruction and myocardial hemorrhage, is higher in individuals bearing the UGT1A9*3 allele [17].

To understand the impact of this genetic variant on ischemia-reperfusion injury more thoroughly, it is necessary to consider the pharmacogenomic and pharmacokinetic characteristics of the *UGT1A9* enzyme. This approach may allow the identification of individualized treatment options. In this sense, medications or genetic changes that influence *UGT1A9* activity are regarded as possible therapeutic targets to lessen the risk of ischemia-reperfusion injury. Moreover, the reduction in the glucuronidation of the cardioprotective endogenous compounds, which results in increased oxidative and inflammatory stress during ischemia-reperfusion injury, indicates that the molecular pathways of the UGT1A9*3 allele should also be elucidated [18,19]. These studies can facilitate the development of personalized cardioprotective methods by shedding light on the contribution of the UGT1A9*3 allele to increased cardiac ischemia sensitivity.

The *UGT1A9* enzyme, particularly in individuals carrying the *3 allele, may exacerbate the severity of cardiac microvascular disease by causing the clearance of endogenous metabolites that are the main players of inflammatory processes to be delayed [20]. These metabolites then cause the production of reactive oxygen species and the occurrence of inflammation in the myocardial tissue. These processes may explain why CK-MB is released from non-myocardial sources, and as a result, troponin levels stay at normal values. Additionally, the decrease in *UGT1A9* activity due to genetic factors may double the risk of statin-induced myopathies in an individual [20].

In addition, reduced glucuronidation of vasoconstrictive and pro-inflammatory eicosanoids such as 20-HETE, resulting from low *UGT1A9* activity, may lead to increased susceptibility to microvascular dysfunction and ischemia-related injury [21]. This condition can contribute to the selective elevation of CK-MB by the prolongation of the inflammatory cascade, thus further extending the injurious process caused by the release of endogenous toxins and subsequent cellular damage [21]. Conversely, the buildup of endogenous substances that are normally metabolized by *UGT1A9* may result in vascular endothelial dysfunction and arterial stiffness, thereby exacerbating coronary artery microvascular dysfunction and increasing myocardial damage [22]. The above mechanism implies that a deficiency in *UGT1A9* glucuronidation capacity leads to an increased inflammatory and oxidative load in the cardiovascular system; thus, myotoxicity introduced by statins is more likely, which may lead to an elevation of CK-MB without changes in troponin levels. The mechanistic considerations mentioned above, for example, the roles of oxidative stress, defective glucuronidation, or drug-induced myotoxicity, should be considered as hypothesis-generating rather than proven explanations. In view of the observational nature of this study and the lack of direct measurements of oxidative stress markers, muscle injury biomarkers other than CK-MB, or detailed drug exposure data, these mechanisms remain unconfirmed. On the contrary, they are suggested to explain the biological plausibility of the dissociation observed between Troponin I and CK-MB levels and to facilitate the next experimental and clinical studies.

The raised CK-MB level, along with normal troponin, indicates that the reduction in statin metabolism caused by the UGT1A9*3 allele has led to the build-up of toxic statin metabolites in muscle cells, which then imparts muscle damage resulting in the release of CK-MB. The mechanism involved here overlaps with the polymorphisms of other drug-metabolizing enzymes, like the extent of increase in CK associated with genetic variants such as CYP3A5*3, thus suggesting the existence of several genetic factors that may predispose to drug-induced myotoxicity [22].

Therefore, it is necessary to account for the interaction of polymorphisms not only for different enzymes but also for the single-nucleotide polymorphism in the *CYP3A5* gene that might have synergistic or contributing roles in the development of statin-induced muscle damage, for example, by [22,23]. In addition, it should be remembered that the enzyme *UGT1A4* is also involved in atorvastatin metabolism, but the influence of its variants on the occurrence of statin-related side effects has not been established yet [21].

In such a case, individuals with reduced *UGT1A9* activity who are also treated with a drug inhibiting UGT enzymes, such as fenofibrate, may experience a further enhancement in the levels of statin lactones that is likely to increase the risk of myotoxicity [21]. This shows that the combined use of drugs should be very carefully evaluated, especially in patients with heart failure, and the *UGT1A* genotype should be considered as one of the most important biomarkers in the statin therapy personalization [24,25]. Differences in the *UGT1A* genes that influence the individual’s reaction to statins serve as a rationale for the implementation of pharmacogenomic testing as a standard in clinical practice [26]. This means that a complete pharmacogenetic panel may be designed to include the prediction of statin exposure and consequent muscle damage risk on an individual basis using the genetic variants of various enzymes that are responsible for drug metabolism [20]. So, the creation of a genetics-based risk score can be instrumental in the timely identification of those most vulnerable to statin-induced myopathy and the optimization of their treatment plans.

Impairment of the *UGT1A9* enzyme activity, especially in persons with the *3 allele, can aggravate the damage of cardiac microvessels by resulting in the slow clearance of endogenous metabolites that are the key players in the inflammatory processes [26]. Consequently, oxidative stress and inflammation in the heart tissue are elevated; thus, the release of CK-MB from non-myocardial sources is increased, and troponin levels remain normal. Moreover, the genetically driven decrease in *UGT1A9* activity, in particular, along with the presence of variants in the SLCO1B1 gene that cause an increase in plasma drug levels, may elevate the risk of statin-induced myopathies [20]. In addition, the lack of glucuronidation of 20-HETE, a vasoconstrictive and pro-inflammatory eicosanoid, due to the low activity of *UGT1A9*, can result in the increased sensitivity to microvascular dysfunction and the consequent ischemia-related injury [21]. Such a case may also help clarify the local release of CK-MB, thus contributing to the prolongation of the inflammatory chain reaction caused by the accumulation of endogenous toxins and the following elevation in cellular damage [21].

Moreover, an overload of endogenous compounds metabolized by *UGT1A9* has the potential to cause vascular endothelial dysfunction as well as arterial stiffness that, in turn, can worsen coronary artery microvascular dysfunction and lead to an increase in myocardial damage [22]. The indicated mechanism implies that a deficiency in *UGT1A9* glucuronidation capacity escalates the inflammation and oxidative stress in the cardiovascular system, thus it is the condition in which statin-induced myotoxicity may occur, and myotoxicity is the cause of CK-MB elevation without concomitant troponin increase.

Variants in genes that affect the ability of tissues to cope with reperfusion injury suggest that the UGT1A9*3 allele may be a factor that results in cardiac ischemia-reperfusion injury to the tissue. The propagation of oxidative stress and inflammatory response after the reperfusion event may be, therefore, more pronounced due to low *UGT1A9* activity, and as a result, the extent of myocardial injury may be increased. A hypothetical explanation could be that diminished glucuronidation of protective endogenous compounds metabolized by *UGT1A9* leads to selective CK-MB elevation through the aggravation of the ischemia-reperfusion injury process, whereas troponin levels remain unaffected. The origin of this case could be due to the occurrence of an insufficient detoxification system of free radicals and pro-inflammatory mediators generated during the reperfusion phase, which eventually causes more cell damage and thus, a selective increase in CK-MB as a non-specific marker of muscle damage [27].

Understanding how the UGT1A9*3 allele elevates the risk of cardiovascular diseases, especially in the case of statin-induced muscle toxicity and ischemia-reperfusion injury, would be clinically valuable. While statin-related myotoxicity could be one reasonable biological process that explains how carriers of the reduced-function UGT1A9 variants isolated CK-MB elevation have, the present study did not have individual statin exposure data. Consequently, any suggestion of an association between the UGT1A9*3 genotype and statin-induced muscle injury would only be a hypothesis worthy of further testing, not a confirmation. It is necessary to investigate in detail the role of impaired drug metabolism and the removal of endogenous toxic compounds in the selective increase of cardiac injury biomarkers in individuals with this genetic variant. Clarifying the different interactions of the *3 allele with cardiac and skeletal muscle based on *UGT1A9*’s substrate specificity and tissue-specific expression profile will provide more insight into the pathophysiological processes. The observation that Troponin I levels were unaffected by *UGT1A9* genotype, while CK-MB showed genotype-associated variability, is consistent with the established specificity and clinical role of contemporary cardiac biomarkers. Importantly, these findings do not challenge current emergency diagnostic algorithms for acute coronary syndrome, which appropriately prioritize troponin as the primary marker of myocardial necrosis. Rather, the potential relevance of *UGT1A9*-associated CK-MB variability may be confined to more specific clinical contexts in which CK-MB is still considered a complementary marker, such as the evaluation of suspected reinfarction after revascularization procedures or in complex clinical scenarios where troponin interpretation may be limited. Within this narrower framework, genetic modulation of CK-MB levels may represent a confounding factor that warrants consideration, rather than a determinant of primary diagnosis.

An important limitation of the present study is the reliance on biomarker variability, particularly CK-MB, as the primary outcome measure. Although CK-MB has historically been used in the assessment of cardiac injury, it represents a surrogate biomarker with limited specificity and is influenced by multiple confounding factors, including skeletal muscle injury, systemic inflammation, renal function, and drug-related effects. Therefore, the observed association between UGT1A9*3 genotype and CK-MB levels should be interpreted cautiously and viewed as hypothesis-generating rather than indicative of direct myocardial damage or clinical outcomes. Future studies incorporating more specific endpoints, longitudinal clinical follow-up, and detailed medication exposure data will be necessary to clarify the clinical significance of these findings.

In this respect, considering the major contribution of myocardial ischemia-reperfusion injury to worldwide morbidity and mortality, it is vital to understand how the UGT1A9*3 allele is involved in this process to be able to develop cardioprotective strategies [28]. At the individual pharmacological level, *UGT1A9* genotyping can result in optimal adjustment of treatment and the avoidance of side effects; therefore, it is mainly advantageous for patients who receive statin therapy or are at high risk of ischemia-reperfusion injury [29,30].

## 4. Materials and Methods

### 4.1. Study Design

The present study was conducted in accordance with the Declaration of Helsinki and was approved by the Ethics Committee of Pamukkale University Faculty of Medicine (approval code: E-60116787-020-789527; approval date: 3 December 2025).

### 4.2. Study Population

The demographic characteristics of the study population reflected the typical profile of patients presenting with acute cardiac symptoms to a tertiary emergency department. A total of 207 patients were included in the final analysis. The mean age of the cohort was consistent with an adult population commonly affected by ischemic and non-ischemic cardiac disorders, with individuals ranging broadly across middle-aged and older age groups. Both male and female patients were represented, reflecting the real-world distribution of cardiovascular presentations in the Emergency Department of Pamukkale University Hospital. Patients were consecutively enrolled between 2019 and 2024 from among all eligible individuals presenting to the emergency department with acute cardiac symptoms during the study period.

Sex distribution indicated that men constituted a slightly higher proportion of the cohort, consistent with the known epidemiology of acute coronary syndromes. The age spectrum and sex balance of the population supported the clinical heterogeneity of cardiac presentations, including myocardial infarction, unstable angina, heart failure exacerbations, and arrhythmia-related symptoms.

Vital parameters recorded at admission, including blood pressure, heart rate, oxygen saturation, and temperature, provided an objective profile of initial clinical severity. Laboratory values such as complete blood count, renal and hepatic function markers, inflammatory markers (CRP, ferritin), coagulation parameters (D-dimer), and cardiac biomarkers (Troponin I and CK-MB) further characterized the physiological status of the patients upon arrival at the emergency department.

Serum cardiac biomarkers were measured using routine clinical laboratory methods at Pamukkale University Hospital. Troponin I levels were determined using a chemiluminescent immunoassay on an automated analyzer (e.g., Abbott Architect i2000SR, Abbott Diagnostics, Abbott Park, IL, USA). CK-MB concentrations were measured using an enzymatic immunoassay on the same automated platform.

### 4.3. Genotyping

The discrepancy in the number of patients analyzed for UGT1A9*3 compared with UGT1A9*1 and *2 was due to unsuccessful or incomplete genotyping for the UGT1A9*3 allele in a subset of samples. Specifically, although all 207 patients were initially included in the study and successfully genotyped for UGT1A9*1 and UGT1A9*2, reliable UGT1A9*3 genotyping results could only be obtained for 172 patients. Samples with insufficient DNA quality or ambiguous amplification signals for the 3 allele were excluded from the UGT1A9*3-specific analyses. Genomic DNA was isolated from whole blood using the Qiagen QIAamp DNA Mini Kit, according to the manufacturer’s instructions [31]. Genotyping of *UGT1A9* polymorphisms (UGT1A9*1, *2, *3) was performed using allele-specific PCR and TaqMan^®^ Real-Time PCR assays, following standard pharmacogenetic protocols. Genotyping of *UGT1A9* polymorphisms (*1, *2, *3) was performed using commercially available TaqMan^®^ SNP Genotyping Assays (Applied Biosystems, Foster City, CA, USA), according to the manufacturer’s instructions. Primer and probe sequences are proprietary information provided by the manufacturer. Standard reaction conditions recommended by the manufacturer were applied, including thermal cycling parameters and reagent concentrations. Appropriate positive and negative controls were included in each run.

### 4.4. Statistical Analysis

All statistical analyses were performed using Python (version 3.9)-based data analysis tools. Continuous variables, including age, Troponin I, CK-MB, CRP, ferritin, D-dimer, and other laboratory markers, were assessed for normality using visual inspection of histograms and quantile–quantile plots. As most variables exhibited non-normal distributions, continuous data were summarized as median and interquartile range (IQR). Categorical variables, including sex, diagnostic categories, and *UGT1A9* genotype groups (*wt/wt*, wt/*3, *3/*3), were presented as absolute numbers and percentages. Hardy–Weinberg equilibrium for genotype distributions was assessed using the chi-square test.

Comparisons of continuous variables across *UGT1A9* genotype groups were performed using the Kruskal–Wallis test, followed by post hoc pairwise Mann–Whitney U tests where appropriate. The association between UGT1A9*3 allelic status and Troponin I or CK-MB levels was evaluated using these nonparametric methods due to skewed distributions. Categorical associations, such as genotype distribution across cardiac diagnostic groups, were examined using the chi-square test of independence. Extreme values were retained, and nonparametric statistical methods were used to minimize the influence of outliers on group comparisons.

Effect sizes for nonparametric comparisons were calculated when applicable. Missing data was handled using listwise deletion. Statistical significance was set at a two-sided *p* < 0.05. The results were visualized using bar plots, box plots, and heatmaps to illustrate genotype distribution, biomarker variability, and diagnosis–genotype relationships.

As the study was of a pilot nature and key laboratory variables were not normally distributed, nonparametric statistical methods were intentionally chosen. The limited number of individuals in some genotype subgroups, especially the UGT1A9*3/*3 group, made it impossible to apply multivariable regression models without the risk of overfitting the model. Consequently, the analyses were based on robust, distribution-free methods for the comparison of genotype-associated differences in cardiac biomarker levels.

Missing data were managed by listwise deletion, and the analyses were carried out only in complete cases. Due to the exploratory nature of this research and the small number of comparisons that were predefined, a formal correction for multiple testing was not implemented. The results of all statistical tests were interpreted cautiously, and the emphasis was placed on the patterns of effects instead of individual *p*-values.

### 4.5. Three-Dimensional Modeling of Wild-Type and Mutant Proteins

The AlphaFold 3 web server was utilized to build the three models of wild-type *UGT1A9* (WT) and the mutated isoforms (C3Y and M33T) [32]. The models were validated with the MolProbity web server of Duke University [33]. The validation was based on the Ramachandran plot outliers and favored rotamer percentages.

## 5. Conclusions

The broad age range of the study population was a possible source of heterogeneity and might have acted as a confounding factor affecting the levels of cardiac biomarkers. Even though it cannot be ruled out that there are age-related differences in the patterns of myocardial injury, the present analysis was not capable of stratifying patients by age due to sample size requirements. As a result, the findings should be considered as representing a mixed emergency department population rather than applying to specific age-defined subgroups.

One limitation of this research is that there were only seven patients homozygous for the UGT1A9*3/*3 genotype, which is a very small sample and could decrease the statistical power of the results for this group. This drawback is due to the very low population frequency of the *3/*3 genotype and is consistent with previous pharmacogenetic studies. So, the *3/*3 group findings should be seen as tentative and exploratory. In any case, the researchers considered that the existence of this group was important to comprehensively depict *UGT1A9* genetic variability and to raise questions that can be addressed in future studies.

This research focused on candidate genes through a gene-targeted study, with a particular emphasis on *UGT1A9*. The gene was chosen due to *UGT1A9*’s major role in glucuronidation pathways involved in statin metabolism and the clearance of endogenous compounds. Although multi-gene pharmacogenetic models can provide a more comprehensive view, single-gene analysis in this case allowed for a more accurate assessment of *UGT1A9*-specific effects on the variability of cardiac biomarkers. However, pharmacogenetically relevant gene interactions, for example, with SLCO1B1 or CYP family members, cannot be ruled out and should be the subject of future multi-gene or polygenic research.

There is one additional limitation of this study worth mentioning: the absence of multivariable adjustment of CK-MB levels for factors that may influence them, for example, age, renal function, inflammatory status, clinical diagnosis, and medication exposure. As there were very few patients in certain genotype subgroups, especially the UGT1A9 *3/*3 group, multivariable modeling was not performed because it would have risked model overfitting. Therefore, the reported relationships should be viewed as merely exploratory and hypothesis-generating. Future research with larger sample sizes and more evenly distributed genotype groups will be required to substantiate these observations using fully adjusted multivariable models.

Such extensive studies highlight the significance of *UGT1A* genes in pharmacogenetic research and provide a novel source of targets for the development of patient-specific treatment strategies and the reduction of drug side effects. In addition, interactions between *UGT1A* proteins and cellular metabolism, as well as the impact of these interactions on detoxification processes, are promising topics for future research. Interactions between the detoxification functions of *UGT1A* enzymes and their effect on overall metabolic cellular functions should be elucidated further through interdisciplinary studies.

## Figures and Tables

**Table 1:** Distribution of *UGT1A9* genotypes in the patient group.

Genotype	Patient Group	Phenotype	Expected Enzyme Activity
UGT1A9*1 wt/wt	113 (54.5)	Normal metabolizer	Normal
UGT1A9*1 wt/*1	94 (45.5)	Intermediate metabolizer	Slightly decreased
UGT1A9*2 wt/wt	144 (69.7)	Normal metabolizer	Normal
UGT1A9*2 wt/*2	63 (30.3)	Intermediate metabolizer	Decreased
UGT1A9*3 wt/wt	109 (64)	Normal metabolizer	Normal
UGT1A9*3 wt/*3	56 (32)	Intermediate metabolizer	Reduced
UGT1A9*3 *3/*3	7 (4)	Poor metabolizer	Significantly reduced

## Data Availability

The original contributions presented in this study are included in the article. Further inquiries can be directed to the corresponding author.

## Abbreviations

ACS	Acute coronary syndrome
CK-MB	Creatine kinase–MB
CRP	C-reactive protein
ED	Emergency department
HWE	Hardy–Weinberg equilibrium
IQR	Interquartile range
NSTEMI	Non–ST elevation myocardial infarction
PCR	Polymerase chain reaction
STEMI	ST-segment elevation myocardial infarction
UGT	Uridine diphosphate glucuronosyltransferase
WT	Wild-type
